# Effect of Severity of Liver Cirrhosis on Surgical Outcomes of Hepatocellular Carcinoma After Liver Resection and Microwave Coagulation

**DOI:** 10.3389/fonc.2021.745615

**Published:** 2021-10-06

**Authors:** Jiang Li, Hai-su Tao, Jian Li, Wen-qiang Wang, Wei-wei Sheng, Zhi-yong Huang, Er-lei Zhang

**Affiliations:** ^1^ Hepatic Surgery Center, Tongji Hospital, Tongji Medical College, Huazhong University of Science and Technology, Wuhan, China; ^2^ Hepatobiliary Surgery, The First Affiliated Hospital, College of Medicine, Shihezi University, Shihezi, China; ^3^ Department of General Surgery, People’s Hospital of Wuning County, Jiujiang, China

**Keywords:** liver cirrhosis, liver resection, percutaneous microwave coagulation therapy, clinical scoring system, prognosis, hepatocellular carcinoma

## Abstract

**Background:**

Liver resection (LR) and percutaneous microwave coagulation therapy (PMCT) are both considered as radical treatments for small hepatocellular carcinoma (HCC). However, it is still unclear whether to select LR or PMCT in HCC patients with different degrees of liver cirrhosis. The purpose of this study was to compare the efficacy of LR and PMCT in the treatment of solitary and small HCC accompanied with different degrees of liver cirrhosis.

**Methods:**

In this study, 230 patients with solitary HCC lesions ≤ 3 cm and Child-Pugh A liver function were retrospectively reviewed. Among these patients, 122 patients underwent LR, and 108 received PMCT. The short- and long-term outcomes were compared between these two procedures. Severity of liver cirrhosis was evaluated by using clinical scoring system (CSS) as previously published. Subgroup analysis based on CSS was performed to evaluate the effect of severity of liver cirrhosis on surgical outcomes after LR and PMCT.

**Results:**

There was no mortality within 90 days in both groups. Major complications were significantly more frequent in the LR group than in the PMCT group (18.8% *vs*. 4.6%, *p*<0.001). However, LR provided better surgical outcomes than PMCT. The 5-year overall survival (OS) rates for the LR and PMCT groups were 65.2% and 42%, respectively (*p*=0.006), and the corresponding disease-free survival (DFS) rates were 51.7% and 31.5%, respectively (*p*=0.004). Nevertheless, subgroup analysis showed that PMCT provided long-term outcomes that were similar to LR and lower surgical complications in HCC patients with CSS score≥4.

**Conclusions:**

LR may provide better OS and DFS rates than PMCT for patients with solitary HCC lesions ≤ 3 cm and Child-Pugh A liver function irrespective of liver cirrhosis. PMCT should be viewed as the optimal treatment for solitary and small HCC with severe cirrhosis.

## Introduction

Hepatocellular carcinoma (HCC) is the fifth most common malignancy in the world (1), and its incidence has been on the rise in recent years (2). Notably, HCC is increasingly detected and diagnosed at an early stage of the disease, however, the selection of optimal surgical treatments for patients with early-stage HCC remains controversial, especially for those with liver cirrhosis. Many guidelines recommend that liver resection (LR) and liver transplantation (LT) are considered as potentially curative therapies for patients with early-stage HCC (3, 4). For HCC patients with severe cirrhosis HCC and within Milan criteria, LT is widely accepted as the gold standard as it could eliminate both the tumors and cirrhotic liver which is prone to *de novo* recurrences of HCC. LT is not a conventional treatment option due to factors such as shortage of donor livers, the high associated cost and disease progression during the waiting period. Therefore, LR is still widely considered as the first option for the treatment of HCC patients without cirrhosis or Child-Pugh class A cirrhosis without portal hypertension (PH) (3, 5). In recent years, local ablations (LA), such as percutaneous microwave coagulation therapy(PMCT) and radiofrequency ablation (RFA), have been recommended as the first-line therapeutic options for patients with early-stage HCC and PMCT seems to show some advantages over RFA regarding efficacy, including better tumor control for perivascular HCCs, and better necrosis rate for cirrhotic liver (6–8). LR may sacrifice additional normal liver tissue which is critical for those with advanced liver cirrhosis, and may lead to severe surgical complications, such as liver failure (9). PMCT is easier to carry out, recovers faster, less invasive, and has a lower rate of liver decompensation in comparison with LR (10, 11). However, the degree of tumor necrosis and the necrosis range of LA are unsatisfactory. Whether LR or PMCT is an optimal option for early-stage HCC with different degrees of liver cirrhosis remains unclear.

Previous studies suggested that the prognosis of HCC depended not only on the treatment strategy and the tumor itself, but also on the underlying liver disease (12), such as chronic hepatitis or liver cirrhosis (1, 4). It has been reported that 60 to 90% of HCC patients in China have underlying liver cirrhosis (13, 14). Unsurprisingly, cirrhosis has been identified as one of the most important risk factors for the development of HCC (15), as well as one of the most important prognostic factors after surgical treatment of HCC (16). The severity of liver cirrhosis was proven to be closely related with the grade of portal hypertension (17), however, liver cirrhosis affected surgical outcomes of HCC independent of portal hypertension (18). Evaluating the status of underlying liver disease only by measuring Child-Pugh liver function and determining the “presence” or “absence” of cirrhosis is obviously unreasonable (14). Our previous studies indicated that the severity of liver cirrhosis significantly influences the short- and long-term surgical outcomes and there existed varied degrees of liver cirrhosis for HCC patients with Child-pugh A liver function and indocyanine green retention test at 15 minutes (ICG-R15) <10% (14, 18–21). Therefore, the severity of liver cirrhosis should be further sub-classified to form a reasonable surgical treatment plan with the aim of decreasing surgical complications and improving surgical outcomes (21).

Liver biopsy is considered as the gold standard for evaluating the severity of cirrhosis (22), but its invasiveness and sampling error preclude its preoperative application in HCC patients. Our previous study developed a clinical scoring system (CSS) as a non-invasive method for sub-classified the severity of liver cirrhosis in HCC patients (as described in **Table 1**) (23). The CSS exhibited high diagnostic accuracy in predicting the severity of cirrhosis, and its accuracy in predicting severe cirrhosis was 85.3%.

**Table 1:** TongJi-clinical scoring model for staging liver cirrhosis.

Clinical variables	Score		
	0	1	2
Varicosity (V1)	none	F1	F2
Portal vein diameter (mm) (V2)	<12	12-14	>14
Spleen thickness (cm) (V3)	<4.0	4.0-5.0	>5.0
Platelet count(109/L) (V4)	≥100	70-100	<70
	Non/mild cirrhosis	Moderate cirrhosis	Severe cirrhosis
clinical scoring system (CSS)	0-1	2-3	≥4

The purpose of this study was to compare the surgical efficacy between LR and PMCT for single HCC tumors ≤ 3cm in patients with Child-Pugh A liver function, and further explore the optimal choice of treatment for small and solitary HCC with different degrees of liver cirrhosis which was pre-operatively evaluated according to the CSS.

## Methods

This study was conducted in accordance with the Helsinki Declaration and approved by the Medical Ethics Committee of Tongji Hospital Affiliated to Huazhong University of Science and Technology. All patients gave written informed consent for post-operative data analysis. A retrospective study was conducted on patients with HCC who received curative treatment in Tongji Hospital, Huazhong University of Science and Technology from January 2008 to December 2014. Patients who met the following criteria were included in the study:

1. The diagnosis of HCC was based on postoperative histopathological examination of the resected specimen or clinical diagnosis according to the American Association for the Study of the Liver Disease (AASLD) criteria (3), including AFP and/or DCP examination in combination with radiographic examination, and was confirmed by two senior physicians.
2. Single nodular HCC tumor with a maximum diameter of 3 cm. There was no portal vein thrombosis or extrahepatic metastasis.
3. Child-Pugh grade A liver function.
4. No previous surgical treatment history of HCC.

The basic information of the HCC patients and preoperative examination results were obtained from the electronic medical record system, including platelet count, aspartate aminotransferase (AST), prothrombin time (PT), albumin, total bilirubin, alpha fetoprotein (AFP), hepatitis B and hepatitis C serological detection, ultrasonography (US), computed tomography (CT), magnetic resonance imaging (MRI).

All treatments followed the clinical guidelines for the treatment of HCC at the time. The decision for LR or PMCT was made based on the disease status (such as tumor location, surgical risk, feasibility of treatment) and the patients’ preferences. Surgical resection was routinely performed using an open method. PMCT was usually performed under ultrasound guidance.

### Surgical Resection Procedure

Surgery was performed under general anesthesia with low central venous pressure (CVP) anesthesia (≤5 mmHg) using a right subcostal incision. We performed non-anatomical partial hepatectomy with more than 0.5 cm tumor-free margin in the cirrhotic patients. Intraoperative ultrasonography was routinely used during surgery to estimate the number, size, location, and boundary of tumors. Cavitron ultrasonic aspiration (CUSA, Valleylab Corp, USA) and Ultrasonic Scalpel (Johnson & Johnson Ltd, USA) devices were used to dissect the liver tissue. In case of accidental bleeding, the Pringle maneuver was routinely conducted, with clamping and unclamping times of 15 and 5 min, respectively.

### PMCT Procedure

HCC was diagnosed by CT and MRI in line with the guidelines of the AASLD (3). PMCT was performed using ECO-100C microwave therapy instruments (ECO CO., LTD, Nanjing, China) with a frequency of 2450-MHz. The PMCT procedure was performed by a professional hepatobiliary surgeon after local anesthesia with 2% lidocaine or general inhalation anesthesia. Real-time ultrasonography (Aloka 5000, ALOKA CO., LTD, Tokyo, Japan) was used to guide and continuously monitor the entire process. After anesthesia was achieved, a 15-cm 16-guage cooling unipolar was inserted into the center of the nodule, and coagulation therapy was performed at 60-80 W output, for 8-10 min per ablation. The ablation was performed repeatedly until the tumors underwent complete necrosis as monitored using real-time ultrasonography, and the hyperechoic area overlapped the area of the tumor with a surrounding ≥1 cm safety margin. Three days later, contrast-enhanced ultrasound was used to judge whether the ablation was successfully completed. If tumor necrosis was considered to be incomplete, PMCT treatment was performed again on the nodules showing incomplete necrosis 1 week after the initial treatment. It should be noted that for a single irregular tumor with a diameter of more than 2.5 cm, a double needle beam can be directly used for ablation at the same time to achieve complete ablation.

### Follow-Up and Efficacy

Liver function, alpha-fetoprotein (AFP), chest radiography and abdominal ultrasonography were routinely monitored every 2-3 months after surgery. Patients with suspected tumor recurrence or metastasis were diagnosed using CT, MR, contrast-enhanced ultrasound, or positron emission tomography (PET). The primary endpoints were overall survival (OS) and disease-free survival (DFS). OS was defined as the time between surgery and the patient’s death or last follow-up. DFS was defined as the time from surgery to recurrence or distant metastasis of HCC.

### Statistical Analysis

SPSS version 25.0 (IBM Corp., USA) was used to analyze the data. Continuous variables were compared using the Mann-Whitney U test, and ordinal variables were compared using Pearson’s χ^2^ test. Median and quartile ranges were applied to data that are not normally distributed. The Mann-Whitney U test was used to assess the significance of differences in laboratory parameters and clinical characteristics between groups. The Kaplan-Meier method was used to generate OS and DFS curves, and the log-rank test was used for comparison. The Cox proportional hazards regression model was applied to calculate the hazard ratio (HR) for survival and the 95% confidence intervals (CI) of prognostic factors for RFS or OS based on the univariate and multivariate analyses. In order to avoid collinearity, the indicators that make up the CSS were not included in the multivariate analysis. Subgroup analysis was performed to assess the effect of CSS on survival. All statistical tests were two-sided, and differences with *p*-values <0.05 were considered statistically significant.

## Results

### Baseline Characteristics

From January 2008 to December 2014, 1022 patients received first-line treatments for HCC consisting of LR or LA. A total of 230 HCC patients who met the inclusion criteria were included in the study. Among these patients, 122 were in the LR group and 108 were in the PMCT group. All the patients had Child-Pugh class A liver function and there was no statistical difference in the baseline characteristics between the two groups. Approximately 88.3% of the patients were male, and most of them had underlying viral hepatitis B. The proportion of cirrhosis in the two groups was similar (89.3 *vs*. 92.6%, *p*= 0.393). The tumor size was also similar (median: 2.9 *vs*. 2.8 cm, *p*=0.250). **Table 2** summarizes the baseline demographic and clinical characteristics.

**Table 2:** Patient demographic and clinical characteristics.

	All patients (n = 230)	LR (n = 122)	PMCT (n = 108)	*P*-value
Age, year	52 (45-58)	51 (44-57)	54 (48-59)	0.055
Male, %	203.0 (88.3)	111.0 (91.0)	92.0 (85.2)	0.173
HBsAg positive, %	218.0 (94.8)	115.0 (94.3)	103.0 (95.4)	0.706
Cirrhosis, %	192.0 (83.5)	109.0 (89.3)	100.0 (92.6)	0.393
A-fetoprotein, ng/ml	34.5 (6.9-225.3)	34.8 (5.9-277.2)	34.5 (7.9-148)	0.920
Tumor size, cm	2.8 (2.6-3.0)	2.9 (2.5-3.0)	2.8 (2.6-3.0)	0.250
Portal hypertension, %	74.0 (32.2)	34.0 (27.9)	40.0 (37.0)	0.137
White blood cell count, x10^9^/mL	4.6 (3.4-5.5)	4.8 (3.5-5.7)	4.4 (3.3-5.3)	0.143
Alanine aminotransferase, U/L	33.0 (24.0-46.0)	31.0 (25.0-43.0)	33.0 (23.0-54.0)	0.286
Aspartate transaminase, U/L	30.0 (26.0-38.3)	29.0 (24.0-36.0)	31.0 (26.0-42.0)	0.090
Albumin, g/L	36.0 (34.9-37.9)	36.4 (35.2-38.3)	35.5 (34.8-37.3)	0.067
Serum bilirubin, mmol/L	13.5 (9.7-18.1)	13.2 (9.7-17.3)	15.0 (9.4-19.5)	0.194
PT, S	13.0 (11.4-14.1)	13.1 (11.6-14.2)	13.0 (11.4-14.1)	0.308
Presence of esophageal varices, %	80.0 (34.8)	37.0 (30.6)	43.0 (39.8)	0.383
Portal vein diameter, mm	1.2 (1.1-1.4)	1.2 (1.1-1.4)	1.2 (1.2-1.3)	0.406
Spleen thickness, cm	4.3 (3.8-4.9)	4.2 (3.7-4.9)	4.5 (3.8-5)	0.116
Platelet count, x10^9^/mL	112.0 (78.8-145.3)	120.0 (83.8-155.0)	105 (72.0-131.8)	0.055

LR, liver resection; PMCT, percutaneous microwave coagulation therapy.

### Long-Term Outcomes of HCC Patients Between the LR and PMCT Groups

**Figure 1:**
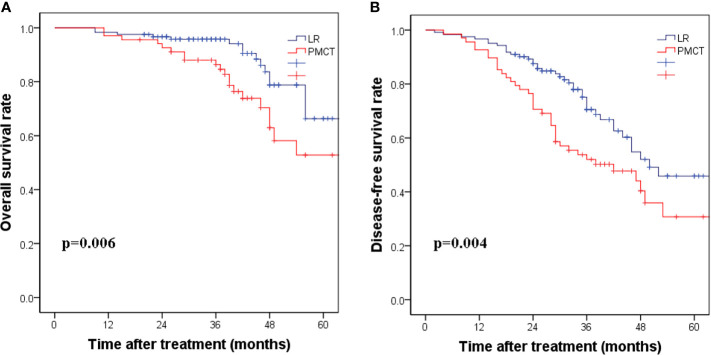
**(A)** The corresponding 1-, 3-, and 5-year disease-free survival rates in the LR group compared with the PMCT group. (*p* = 0.004). **(B)** The corresponding 1-, 3-, and 5-year overall survival rates in the LR group compared with the PMCT group. (*p* = 0.006). LR, liver resection; PMCT, percutaneous microwave coagulation therapy.

In five of the patients who underwent PMCT, the effect was unsatisfactory at the first time, and the PMCT was performed again a week later. A total of 93 patients developed recurrence over a median follow-up period of 46.3 months. In the LR group, 89 patients developed recurrence, with a median follow-up period of 62.3 months. According to the Kaplan-Meier curve analysis, patients who received LR showed better OS and DFS than those who received PMCT. The corresponding 1-, 3-, and 5-year DFS rates were 95.4%, 74.5%, and 51.7% in the LR group, compared with 83.4%, 51.2%, and 31.5% in the PMCT group, respectively. (*p*=0.004, **Figure 1A**). The 1-, 3-, and 5-year OS rates were 97.2%, 91.6%, and 65.2% in the LR group, compared with 90.1%, 72.4%, and 42% in the PMCT group, respectively (*p*=0.006, **Figure 1B**).

### Univariate and Multivariate Analyses of OS and DFS

Based on a univariate analysis, the treatment method, albumin, serum bilirubin, platelet count, portal vein diameter, spleen thickness, varicose veins, cirrhosis and CSS score were associated with DFS (*p* < 0.05, **Table 3**).

**Table 3:** Univariate analysis of prognostic factors of disease-free survival rate.

Factors	Univariate Cox regression		Multivariate Cox regression	
	Hazard ratio (95% CI)	*P* value	Hazard ratio (95% CI)	*P* value
Age (>50,≤50)	1.005 (0.982-1.027)	0.687		
Gender (male, female)	1.031 (0.533-1.994)	0.928		
Treatment method	0.575 (0.376-0.882)	0.011	0.747 (0.481-1.161)	0.195
HBsAg (positive, negative)	1.546 (0.633-3.773)	0.339		
AFP (>400ng/ml,≤400ng/ml)	1.185 (0.773-1.816)	0.436		
White blood cell count (≤3.5x109/mL,>3.5x109/mL)	0.742 (0.478-1.151)	0.183		
Prothrombin time (≤14.5S,>14.5S)	0.924 (0.814-1.049)	0.220		
Alanine aminotransferase (>40U/L,≤40U/L)	0.993 (0.983-1.002)	0.129		
Aspartate transaminase (>40U/L,≤40U/L)	0.997 (0.982-1.013)	0.726		
Albumin (>35g/L,≤35g/L)	0.419 (0.202-0.873)	0.020	1.238 (0.545-2.815)	0.610
Serum bilirubin (>17g/dL,≤17g/dL)	7.358 (3.775-14.342)	0.001	2.114 (1.076-4.155)	0.030
Platelet count (<100/uL, ≥100)	0.401 (0.264-0.611)	0.001		
Portal vein diameter (≤1.2cm,>1.2cm)	2.347 (1.541-3.575)	0.001		
Spleen thickness (≤4cm,>4cm)	1.656 (1.006-2.726)	0.047		
Tumor size, cm	0.819 (0.451-1.488)	0.512		
Portal hypertension (yes, no)	0.989 (0.580-1.688)	0.969		
Presence of esophageal varices (yes, no)	26.445 (9.490-73.693)	0.001		
Cirrhosis (Severe, Mild+Moderate)	17.012 (6.132-47.196)	0.001	3.022 (1.132-8.064)	0.027
CSS (≥4,<4)	2.907 (1.714-4.928)	0.001	4.570 (1.499-13.934)	0.008

In multivariate analysis, serum bilirubin (HR=2.114, 95% CI: 1.076-4.155, *p* < 0.05), cirrhosis (HR=3.022, 95% CI:1.132-8.064, *p* < 0.05) and CSS (HR=4.570, 95% CI: 1.499-13.934, *p* < 0.01) were independent risk factors for DFS. According to the univariate analysis, the treatment method, albumin, serum bilirubin, platelet count, portal vein diameter, spleen thickness, varicose veins, cirrhosis and CSS were associated with OS (*p* < 0.05, **Table 4**). According to the multivariate analysis, independent prognostic factors for OS included serum bilirubin (HR=1.047, 95% CI: 1.005-1.091, *p* < 0.05), cirrhosis (HR=3.746, 95% CI: 1.221-11.495, P < 0.05) and CSS (HR=10.119, 95% CI: 1.706-60.026, *p* < 0.05).

**Table 4:** Univariate analysis of prognostic factors of overall survival rate.

Factors	Univariate Cox regression		Multivariate Cox regression	
	Hazard ratio (95% CI)	*P* value	Hazard ratio (95% CI)	*P* value
Age (>50,≤50)	1.005 (0.981-1.030)	0.702		
Gender (male, female)	1.570 (0.630-3.911)	0.333		
Treatment method	0.594 (0.365-0.964)	0.035	0.814 (0.499-1.327)	0.408
HBsAg (positive, negative)	1.582 (0.638-3.923)	0.332		
AFP (>400ng/ml,≤400ng/ml)	1.199 (0.738-1.949)	0.463		
White blood cell count (≤3.5x109/mL,>3.5x109/mL)	0.936 (0.563-1.556)	0.799		
Prothrombin time (≤14.5S,>14.5S)	0.635 (0.288-1.401)	0.261		
Alanine aminotransferase (>40U/L,≤40U/L)	0.996 (0.985-1.006)	0.428		
Aspartate transaminase (>40U/L,≤40U/L)	1.000 (0.981-1.020)	0.974		
Albumin (>35g/L,≤35g/L)	0.336 (0.135-0.834)	0.019	1.059 (0.403-2.782)	0.907
Serum bilirubin (>17g/dL,≤17g/dL)	11.603 (5.019-26.825)	0.001	1.047 (1.005-1.091)	0.027
Platelet count (<100/uL,≥100)	0.990 (0.984-0.995)	0.001		
Portal vein diameter (≤1.2cm,>1.2cm)	9.100 (2.892-28.631)	0.001		
Spleen thickness (≤4cm,>4cm)	1.466 (1.139-1.885)	0.003		
Tumor size, cm	0.800 (0.404-1.588)	0.542		
Portal hypertension (yes, no)	0.989 (0.580-1.688)	0.270		
Presence of esophageal varices (yes, no)	13.328 (5.349-33.213)	0.001		
Cirrhosis (Severe, Mild+Moderate)	24.519 (9.864-60.948)	0.001	3.746 (1.221-11.495)	0.021
CSS (≥4, <4)	53.492 (13.090-218.605)	0.001	10.119 (1.706-60.026)	0.011

### Subgroup Analysis

**Figure 2:**
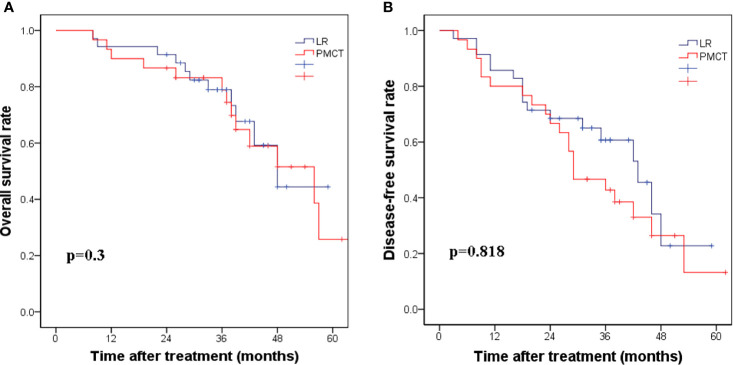
**(A)** The corresponding 1-, 3-, and 5-year disease-free survival rates in the LR group compared with the PMCT group. (*p* = 0.818). **(B)** The corresponding 1-, 3-, and 5-year overall survival rates in the LR group compared with the PMCT group. (*p* = 0.3). LR, liver resection; PMCT, percutaneous microwave coagulation therapy.

Further analysis of subgroups according to the degree of cirrhosis based on CSS showed that when the CSS score ≥4, the corresponding 5-year DFS rates were 22.8% and 16.2%, respectively (*p*=0.818, **Figure 2A**). The 5-year OS rates for the LR group and PMCT group were 41.4% and 25.8%, respectively (*p*=0.3, **Figure 2B**). Thus, there were no significant differences in the DFS and OS rates between the two groups.

### Complications

There was no mortality in both groups during the initial 90 days post-intervention. Hepatic hemorrhage (10/122 *vs*. 0/108) and pain (58/122 *vs*. 13/108) were significantly higher in the LR group than in the PMCT group (*p*<0.05). Pleural effusion (9/122 *vs*. 1/108) and ascites (15/122 *vs*. 5/108) were significantly more common in the LR group (*p*<0.05). Serious complications, such as liver abscess, intraperitoneal bleeding, hepatic infarction, and biliary peritonitis did not occur. Major complications were significantly more frequent in the LR group than in the PMCT group (18.8% *vs*. 4.6%, *p*<0.001). Among HCC patients with the CSS score ≥4, the rate of major complications was 28.4% in the LR group and 5.9% in the PMCT group (*p*=0.001, **Supplemental Figure 1**). The length of hospital stay in the LR group was greater than that in the PMCT group, and the total hospitalization cost was significantly higher than that in the PMCT group.

## Discussion

Nowadays, with the widely use of surveillance programs for HCC in high-risk populations, patients are increasingly diagnosed as early-stage HCC. LR and heat-based local ablations (such as RFA and PMCT) are considered to be the best curative treatments for small HCC in Western countries (10, 24). LR is the most common method in clinical practice, but the resulting tissue trauma is relatively large, and RFA is certainly less invasive. Five randomized controlled trials compared the surgical efficacy of LR and RFA, but they reported conflicting results (25–29). Of them, three randomized controlled trials showed similar survival rates between LR and RFA (25, 27, 29), but in the other two studies, LR was more favorable than RFA in terms of OS and DFS (26, 28). PMCT and RFA have been recommended as first-line therapeutic options for patients with early-stage HCC and limited liver function or severe liver cirrhosis. However, it remains unclear whether MWA is superior to RFA in terms of RFS and OS for the treatment of small and solitary HCC (30, 31). In recent years, PMCT seemed to show some advantages over RFA regarding efficacy, including better tumor control for perivascular HCCs (6), and better necrosis rate for cirrhotic liver (7). Meanwhile, PMCT can overcome the “heat sink effect” which is an obvious disadvantage of RFA (32). A Meta-Analysis of randomized controlled trials indicated a similar efficacy and safety profile between PMCT and RFA, however, distant recurrence rate was significantly lower with PMCT (RR=0.60, 0.39–0.92) in comparison with RFA (8). In China, more than 80% of HCC patients are associated with varied degrees of liver cirrhosis (14). For HCC patients with advanced liver cirrhosis, preserving adequate liver tissue is more important for surgical outcomes, therefore, the authors should consider the effect of liver cirrhosis on tumor necrosis when performed local ablation. A recent study compared the efficacy of RFA and PMCT in achieving complete response in cirrhotic patients with early and very early HCC. The results indicated that PMCT was more efficient than RFA in achieving complete response in HCC nodules with 21 to 35 mm diameter in cirrhotic liver (recurrence rates of RFA and PMCT were 31.9% *versus* 13.5%, *p* = 0.019) (7). Potential reasons may be that PMCT induces higher temperature around the tumor in cirrhotic liver tissues (33), and RFA may result in a cold zone easily due to the slow warming of the target area when the ablative region is restricted to perivascular tissue (6). A recent study included a total of 144 eligible patients with small (≤ 3cm) and solitary perivascular (proximity to hepatic and portal veins) HCC who underwent RFA (N=70) or PMCT (N=74) and compared the therapeutic outcomes of these two treatments (6). The results indicated that a significantly higher local tumor progression rate was observed in the RFA group than the PMCT group (5-year local tumor progression rate: RFA 24.3% *vs*. PMCT 8.4%, *p*=0.030) (6). As the PMCT treatment showing more superiority, in this study, we compared the efficacy of LR and PMCT in early-stage HCC patients. Many centers have adopted LR as the first-line treatment of early-stage HCC. LR can not only remove the tumor but can also remove the blood vessels and the adjacent liver tissue invaded by the tumor, thus helping prevent recurrence and metastasis in the short term. LR is also the preferred method to the treatment of tumors on the surface or margin of the liver (34). However, LR sacrifices more healthy liver tissue and leads to more surgical complications for those with severe liver cirrhosis, requiring longer hospital stay and thus incurring higher costs (29). PMCT is increasingly accepted for the treatment of early-stage HCC due to its convenient operation, less invasiveness, low postoperative liver decompensation, quick recovery and short hospital stay (35). Previous studies suggested that PMCT had comparable surgical outcomes to LR for early-stage HCC patients, with a lower incidence of complications, cheaper medical cost and shorter hospital stay (10, 33). For HCC lesions with a size <3cm, Shi et al. (36) found that PMCT had similar surgical results to LR. However, some scholars also believe that LR offers significantly better 3-year OS and 5-year DFS than PMCT (35). Moreover, larger liver tumors (including metastases) may be more suitable for LR (34), because PMCT treatment requires multiple overlapping ablations, increasing the difficulty of treatment, so that the resulting ablation margin is not as accurate as resection (36). A recent study compared the therapeutic efficacy between the robot-assisted LR and PMCT. After propensity score matching, 3-year recurrence-free survival, OS and cancer-specific survival of the patients in both groups were comparable. PMCT showed advantages of less operation time, less blood loss and lower medical cost, while LR performed better in postoperative liver function. There was no significant difference in incidence of severe complications between two groups (10). Therefore, distinguishing HCC patients who would benefit most from the two treatments will be a crucial clinical challenge in the future.

In previous studies, worse liver function, liver cirrhosis and portal hypertension were considered as important comorbidities that makes HCC patients favorable candidates for PMCT, because surgical resection may cause more blood loss and more severe complications, such as liver failure (33, 37). A study by Chong et al. (37) evaluated the surgical outcomes of HCC patients treated with LR or PMCT and the role of Albumin-Bilirubin (ALBI) score in selecting surgical modalities. Propensity scoring matching analysis according to the ALBI grade was performed. LR offered better overall and disease-free survivals in patients with ALBI grade 1 whereas PMCT provided a significantly better overall survival *(p*=0.025) and a trend towards better disease-free survival (*p*=0.39) in patients with ALBI grade 2 or 3 (37). This study suggested the liver reserve function played a crucial role in the selection of LR or PMCT for early-stage HCC patients. Our previous study indicated that LR may provide better disease-free survival and lower recurrence rates than PMCT for single HCC ≤3 cm and Child-Pugh A cirrhosis, however, PMCT may provide therapeutic effects that are similar to LR for HCC patients with portal hypertension (33). In this study, portal hypertension was defined by an indirect predictor which was less accurate. Furthermore, we did not evaluate the importance of underlying cirrhosis. The severity of liver cirrhosis played an important role in the selection of surgical modality for HCC patients (14). Obvious histological variability in the same stage of liver cirrhosis exists in the majority of HCC patients. Moreover, clinical severity and prognosis within the same histological cirrhosis vary widely, and the risk and surgical outcomes of LR may be different (38). For example, there are still some advanced cirrhosis (F4B-F4C using Laennec scoring system) for compensated cirrhosis or Child-Pugh A cirrhosis; therefore, it should be emphasized these differences to make proper surgical decisions in clinical practice. Unfortunately, it is still unclear whether LR or PMCT is the better treatment option for a single small HCC tumor with different degrees of liver cirrhosis.

In this retrospective study, we found that patients with a single HCC tumor ≤3cm and Child-Pugh class A liver function had longer DFS and OS with LR than with PMCT. At the same time, univariate and multivariate analyses also confirmed that liver cirrhosis was an independent critical risk factor for OS and DFS. In our study, about 83.5% of HCC patients had liver cirrhosis, while previous study had shown that HCC patients with liver cirrhosis had significantly worse long-term outcomes after surgical resection than patients without cirrhosis (39). It is increasingly being recognized that the presence of underlying cirrhosis is closely related to the recurrence of HCC after resection (40). Therefore, the underlying cirrhosis should be considered as a key factor affecting the surgical outcomes (14). Special attention should be paid to assessing the severity of cirrhosis before treatment decisions are made, and there are no recommendations for the treatment of single and small HCC in patients with different degrees of liver cirrhosis. In the clinical settings, the severity of liver cirrhosis may be accurately evaluated pre-operation by liver biopsy, however, the clinical feasibility of this method was severely limited owing to its invasiveness which prevented the collection of histologic information from all the HCC patients at any given time. Thus, there is an urgent need to find a non-invasive method to sub-classify liver cirrhosis that could faithfully evaluates the degrees of liver cirrhosis and that is also essential for the individualization of surgical modalities. By using several simple clinical parameters, we developed a new clinical scoring system to predict the histological severity of cirrhosis with a diagnostic accuracy of more than 80%, especially for those with severe liver cirrhosis (85.3%) (23). The preoperative evaluation of the degrees of liver cirrhosis using this non-invasive method may provide us a strong evidence for the selection of surgical treatments.

Combined with this clinical scoring system, we found that when the score ≥4, the OS and DFS between the two groups was similar. However, the occurrence of surgical complications was more frequent in the LR group than those in the PMCT group (18.8% *vs*. 4.6%, *p*<0.001). PMCT is a relatively less invasive procedure that preservers more liver parenchyma, has a lower cost, shorter hospital stay, and better reproducibility, with a lower risk of postoperative complications. For HCC patients with advanced liver cirrhosis, preserving adequate liver tissue is more important for surgical outcomes. LR may result in increased liver function impairment, increased risk of postoperative liver decompensation and death, and therefore higher costs and longer hospital stays due to the complicated surgical procedure and the sacrifice of more non-neoplastic liver parenchyma (25, 41). It should be pointed out that PMCT-treated HCC patients had worse clinical presentations than those treated with LR although there is no statistical difference between the two groups, therefore, PMCT should be considered as the preferred option, and priority can be given to HCC patients who were contraindicated for surgery due to age, comorbidities or background liver damage. According to the results of this study, PMCT is therefore a better treatment option for HCC patients with severe cirrhosis and tumor size ≤ 3 cm, but we should also consider factors such as the location of the tumor, the experience and proficiency of the operator when choosing PMCT.

Finally, several limitations should be considered in this study. Firstly, due to the small total sample size, the statistical power was low and there is a relatively high possibility of selection bias. Secondly, most of the patients in this study had underlying cirrhosis due to hepatitis B, which is clearly not representative of all HCC patients. Thirdly, some PMCT patients had no pathohistological results, so the well-known factors affecting prognosis such as the degree of tumor differentiation and microvascular invasion could not be analyzed. Lastly, this study was a retrospective single-center study, and multi-center clinical trials should be conducted in the later stage to provide stronger evidence and obtain more accurate results.

In conclusion, this retrospective study shows that LR provides better OS and better DFS than PMCT in patients with a single HCC ≤3cm and Child-Pugh class A liver function irrespective of liver cirrhosis. However, for those patients accompanied with severe liver cirrhosis, PMCT may provide a therapeutic effect similar to that of LR and may represent an optimal option, with less invasive, lower medical cost and complications, which should be considered as a superior option. Large number and multicenter randomized controlled trials should be performed to verify our conclusions in the future.

## Data Availability Statement

The raw data supporting the conclusions of this article will be made available by the authors, without undue reservation.

## Ethics Statement

The studies involving human participants were reviewed and approved by Medical Ethics Committee of Tongji Hospital Affiliated to Huazhong University of Science and Technology. Written informed consent for participation was not required for this study in accordance with the national legislation and the institutional requirements.

## Author Contributions

E-lZ made the concept and design. JiangL, H-sT, JianL, and W-qW acquired and analyzed the data. JiangL drafted the manuscript. E-lZ critically revised the manuscript for the final version. W-wS and JiangL conducted the statistical analysis. All authors contributed to the article and approved the submitted version.

## Funding

This work was supported by the grants from National Natural Science Foundation of China (81902839).

## Conflict of Interest

The authors declare that the research was conducted in the absence of any commercial or financial relationships that could be construed as a potential conflict of interest.

## Publisher’s Note

All claims expressed in this article are solely those of the authors and do not necessarily represent those of their affiliated organizations, or those of the publisher, the editors and the reviewers. Any product that may be evaluated in this article, or claim that may be made by its manufacturer, is not guaranteed or endorsed by the publisher.
