# Supplementary figures and images for: Effect of Severity of Liver Cirrhosis on Surgical Outcomes of Hepatocellular Carcinoma After Liver Resection and Microwave Coagulation
Source: Front Oncol. 2021 Oct 6;11:745615. doi: 10.3389/fonc.2021.745615 (PMC8526975; doi:10.3389/fonc.2021.745615)

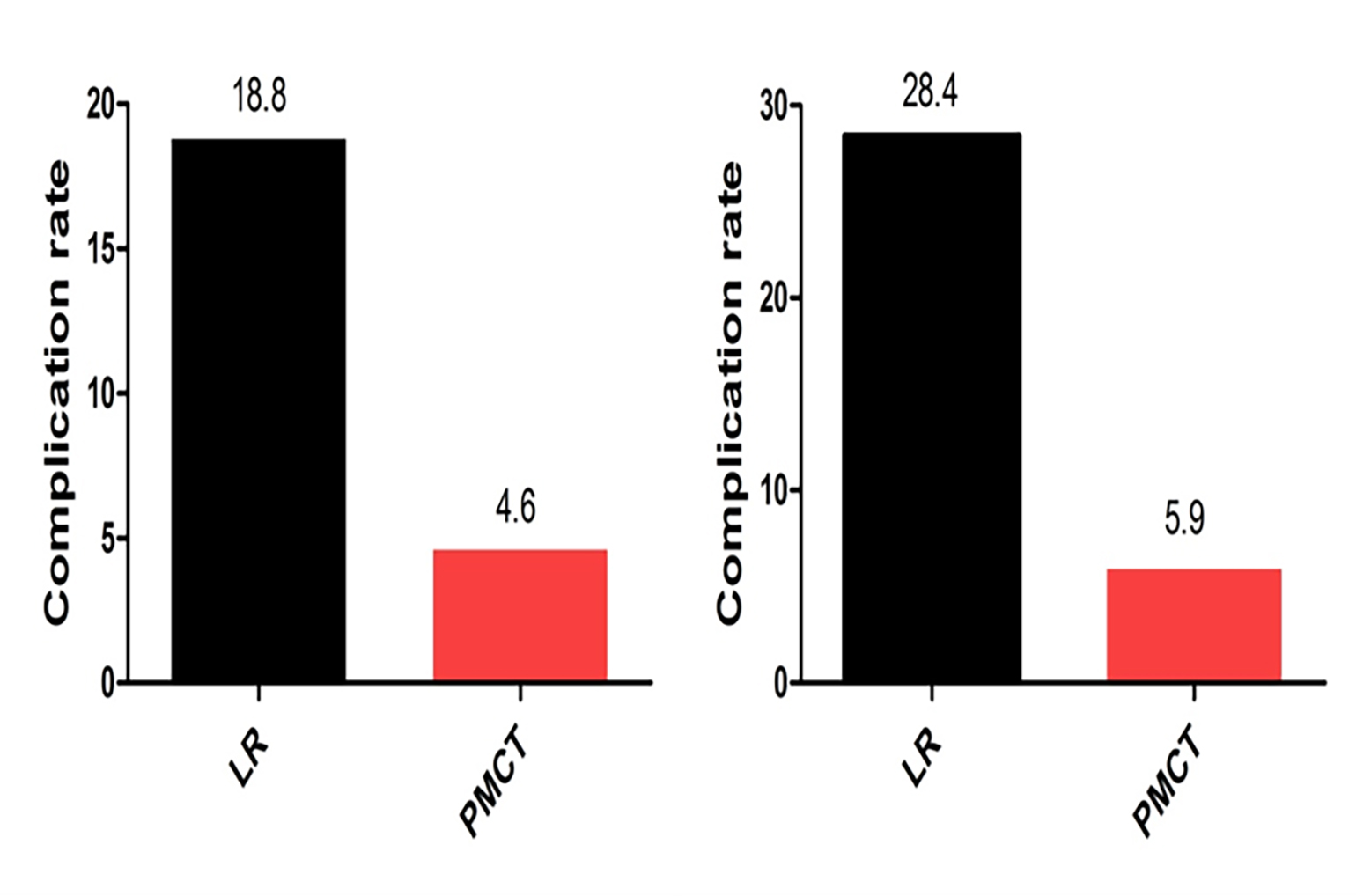

Supplement: Supplementary file 1 [file Image_1.jpeg]
